# Dual U-Net with multi-task attention for automated eyelid curvature quantification

**DOI:** 10.3389/fmed.2025.1631468

**Published:** 2025-07-17

**Authors:** Jimei Wu, Yang Yang, Cheng Wan, Meina Yang, Weihua Yang, Wei Chi

**Affiliations:** ^1^College of Electronic and Information Engineering, Nanjing University of Aeronautics and Astronautics, Nanjing, China; ^2^College of Artificial Intelligence, Nanjing University of Aeronautics and Astronautics, Nanjing, China; ^3^Shenzhen Eye Hospital, Shenzhen Eye Medical Center, Southern Medical University, Shenzhen, China

**Keywords:** eyelid curvature, measurement, palpebral fissure segmentation, corneal segmentation, artificial intelligence

## Abstract

**Objective:**

Eyelid curvature analysis serves as a key morphological indicator in the diagnosis of ophthalmic diseases and postoperative evaluation. This study aims to develop an automated and reproducible image processing method to accurately extract eyelid margin curves from anterior segment images and perform quantitative curvature analysis.

**Methods:**

A dual-branch U-Net architecture is proposed, utilizing a shared encoder and task-specific decoders to simultaneously segment the palpebral fissure and corneal regions. Based on the segmentation results, eyelid margin curves were extracted and fitted with second-order polynomials to calculate curvature values.

**Results:**

A total of 130 anterior segment images were collected. In segmentation tasks, the proposed AtDU-Net model achieved an IoU of 0.979 and a Dice coefficient of 0.989. The automatically measured eyelid curvatures showed high consistency with manual annotations, with correlation coefficients of 0.9032 for the upper eyelid and 0.9154 for the lower eyelid. Bland-Altman analysis indicated that over 92% of the samples fell within the limits of agreement, validating the consistency and reliability of the measurements.

**Conclusion:**

The proposed method demonstrates superior performance in terms of accuracy, robustness, and consistency with manual measurements. It shows strong potential for clinical applications, providing reliable technical support for eyelid morphological analysis and surgical planning.

## 1 Introduction

The eye is a complex and delicate sensory organ, serving as a crucial bridge for perceiving light and color. Its structure can be divided into two main parts: the ocular surface (1) and the fundus (2). The fundus, as the core area for visual signal conversion and transmission, contains structures such as the retina (3) and optic disc (4), which are critical for transforming light signals into neural signals. The ocular surface, on the other hand, mainly consists of the cornea (5) and eyelids (6), playing a vital role in protecting the eye and initially regulating light. The cornea, located on the anterior wall of the eye, is a highly transparent thin membrane that plays a key role in refracting and focusing light during the visual imaging process. Its curvature and transparency directly determine the quality of the image, and its morphological characteristics are closely related to its health status. The eyelid, another essential component of the ocular surface, is mainly divided into the upper eyelid and lower eyelid, which cover the upper and lower portions of the eye, respectively, and serve to protect the eyeball (7). Meibomian gland (MG), large sebaceous glands located in the upper and lower eyelid (8). They are responsible for producing and secreting lipids called blepharoplasts, which constitute the lipid layer of the tear film (9). The upper eyelid is a soft tissue structure that covers the upper part of the eyeball, with its primary functions being to shield the eye from external stimuli and to secrete tears through blinking, keeping the ocular surface moist. The lower eyelid, a curtain-like structure covering the lower part of the palpebral fissure, works in conjunction with the upper eyelid to form a protective barrier for the eyeball. The palpebral fissure refers to the natural opening or gap between the upper and lower eyelids. Its geometric characteristics, including size, shape, and curvature, are critical for ophthalmic diagnosis and functional evaluation of the eye. Abnormalities of the eyelids can occur in various degrees across different ophthalmic diseases (10).

Eyelid abnormalities can cause changes in the curvature of the eyelid. Diseases such as ptosis, Graves’ ophthalmopathy, and eyelid tumors can all result in eyelid abnormalities (11). For instance, ptosis may be associated with brain tumors or autoimmune diseases such as myasthenia gravis. And the clinical manifestations of Thyroid-associated ophthalmopathy are diverse and complex, including unilateral or bilateral eyelid retraction (12). Blepharoptosis, a common indication for upper eyelid surgery, may have a myogenic, neurogenic, traumatic, or mechanical cause (13). In Graves’ orbitopathy, patients may experience eyelid retraction, causing the curvature of the upper eyelid to become more pronounced, with the highest point of the eyelid contour shifting outward, thereby increasing the area of upper temporal region (14). In cases of congenital ptosis (15), the eyelid contour tends to become relatively flattened, with the highest point shifting inward, resulting in a reduction in the area of the upper eyelid region. Additionally, due to abnormalities in the orbicularis oculi muscle and levator tendon membranes, patients with blepharochalasis (16) may exhibit enlarged and thickened upper eyelids, further affecting the curvature of the eyelid contour. Meanwhile, in the field of medical esthetics, eyelid plastic surgery is a common procedure in cosmetic surgery. To create a more refined appearance of the eyes, the curvatures of the upper and lower eyelids have gained increasing attention.

Therefore, the quantitative measurement of eyelid curvature can reflect the morphological changes in the eyelid contour, providing crucial support for ophthalmologists in diagnosing ocular diseases.

Currently, most measurements of eyelid line curvature rely on specialized instruments and physicians. Various metrics, such as margin–reflex distance 1 (MRD1), MRD2, palpebral fissure height (PFH), and eyelid length, are currently being used to objectively assess the shape and condition of the eyelids (17, 18). The concept of the mid-pupil lid distance (MPLD) has been used in various studies to compare the curvature of the eyelid between different patients (19–22). Several studies have explored the measurement of eyelid curvature. For example, Cruz et al. (23) studied palpebral fissure images from 29 patients with Graves’ orbitopathy, 22 patients with congenital ptosis, and 50 healthy individuals without any medical history. They processed these images using NIH Image 1.55 software to extract curvature data for the upper eyelid. By fitting the upper eyelid contour with a second-order polynomial, they obtained the corresponding curvature values. Additionally, Cruz (24) conducted quantitative analyses of the palpebral fissure shapes of 20 severe congenital ptosis cases through cross-sectional digital image processing. This analysis evaluated the curvature of the upper and lower eyelid lines, providing guidance for contour adjustments during surgery, enabling doctors to achieve more precise lateral displacements and optimize surgical outcomes. Maseedupally (25) et al. input uniformly cropped ocular surface images into the i-Metrics software, where professional ophthalmologists manually annotated the eyelid lines. The curvature was then quantified through polynomial fitting. Meanwhile, Malbouisson (26) used a camera with an electro-coupled device to capture palpebral fissure images of 110 healthy subjects and processed these images using NIH Image software on a Macintosh computer, employing a second-order polynomial to fit the contours of the upper and lower eyelids. Garcia (27) quantitatively analyzed the lower eyelid contours of patients with Graves’ orbitopathy, using ImageJ software to adjust the Bezier curves of the lower eyelid for 41 patients and 43 healthy controls. Bezier curves, widely used in computer graphics, define a curve by a mathematical formula and have been suggested to analyze eyelid curvature (28).

However, there are several problems with existing methods for measuring eyelid line curvature. Firstly, although current software can conveniently extract eyelid contour points, professional ophthalmologists are still required for evaluation, which is time-consuming and labor-intensive. Furthermore, when selecting points on the eyelid contour to fit curves for the upper and lower eyelid lines, the number and specific location of these points are easily influenced by the operator’s subjective judgment. This is particularly relevant in medical image segmentation, where labels are often highly subjective (29). In addition, after upper and lower eyelid surgeries, doctors need to dynamically monitor eyelid morphology, and frequent manual measurements are difficult to implement when the number of patients is large. With the continuous improvement of computer technology and data processing capabilities, the development and application of AI are becoming increasingly widespread and in-depth (30). Deep learning algorithms effectively recognize meaningful patterns in images and have been shown to extract pathologic features in medical imaging (31–33). To address this issue, this paper proposes a new method for measuring eyelid curvature based on U-Net. By improving the existing segmentation network, the method automatically segments the palpebral fissure and corneal areas. It then combines traditional image processing and mathematical methods to quantitatively measure the eyelid line curvature. The entire process only requires acquiring the patient’s ocular surface images, enabling the patient to conveniently, quickly, and accurately obtain their eyelid curvature value and promptly understand their eyelid contour condition.

## 2 Materials and methods

### 2.1 Data

This study utilized 130 ocular surface images provided by the Shenzhen Eye Hospital. Considering the dependency of deep learning models on sufficient training data, further dividing the dataset to create a validation set would have significantly reduced the amount of training data, thereby impairing the learning performance of the model. Therefore, we selected 100 images for training and 30 images for testing. Although a separate validation set was not established, the model performance was evaluated on the test set after each training epoch to monitor generalization capability and prevent overfitting. The original image size was 2,974 × 1,984, with an actual width of 14.65 cm and height of 9.77 cm, where each pixel corresponds to 0.04924 mm. The corneal horizontal diameter was 11.5 mm in reality, and through image processing, it was calculated as 954 pixels, corresponding to a corneal horizontal diameter of 0.01205 mm per pixel. To ensure consistent scale and reduce computational cost, the images were resized to 744 × 496. Data augmentation strategies, including random cropping and random horizontal and vertical flipping, were applied to enhance the diversity of the training samples and improve model robustness. The processed training set was then used to accurately segment the palpebral fissure and corneal regions, enabling the measurement of eyelid line curvature.

The data anonymization for corneal surface images was applied before the study, ethical review and approval was not required for the study on human participants in accordance with the local legislation and the institutional requirements. Written informed consent from the patients was not required to participate in this study in accordance with the national legislation and the institutional requirements.

### 2.2 Method

**Figure 1:**
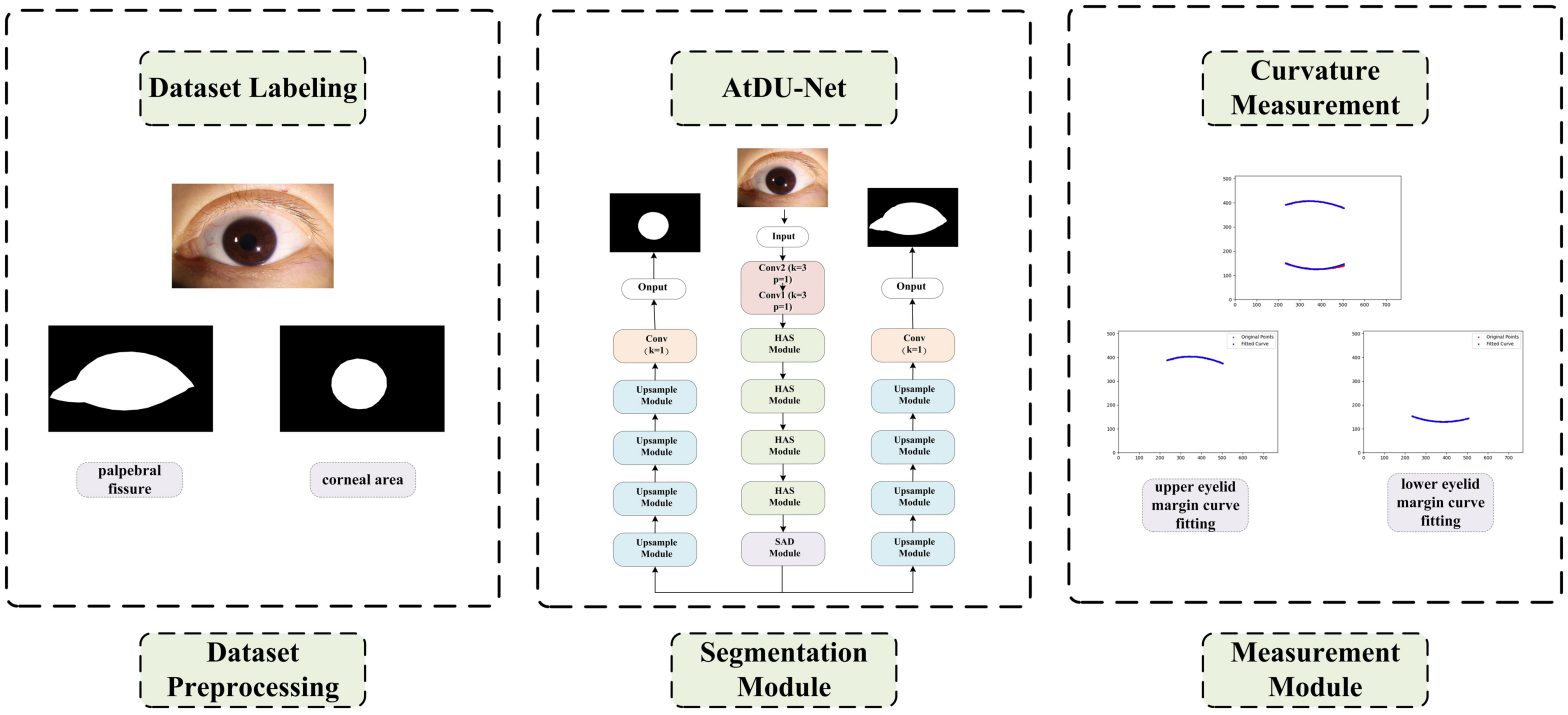
Structure of the automatic eyelid curvature measurement system.

This study has developed a deep learning-based eyelid curvature measurement system, the overall architecture of which is shown in Figure 1. The system consists of three main modules: the dataset preprocessing module, the eyelid-cornea segmentation module, and the curvature measurement module. The aim is to achieve automated measurement of eyelid curvature from ocular image processing, providing efficient and accurate technical support for ophthalmic diagnosis. Firstly, the dataset preprocessing module is responsible for generating high-quality training datasets and standardizing the data to ensure the robustness and generalization ability of the subsequent deep learning models. Secondly, the eyelid-cornea segmentation module uses an improved Attention Double U-Net (AtDU-Net) architecture, combining multi-scale feature extraction with attention mechanisms to achieve precise segmentation of the eyelid and corneal regions. Finally, the curvature measurement module analyzes the segmentation results, extracting the upper and lower eyelid curve shapes and accurately calculating their curvature parameters. Through the efficient collaboration of these three modules, the system achieves the automation of eyelid curvature measurement. It not only improves the accuracy of segmentation and the efficiency of measurement but also provides a reliable basis for early screening of ophthalmic diseases and surgical planning.

#### 2.2.1 Dual U-net architecture

With its encoder-decoder architecture at its core, the U-Net network captures multi-scale global features during the encoder phase, while the decoder phase progressively restores image details. By incorporating skip connections, it retains key spatial information, making it perform exceptionally well in medical image segmentation tasks, especially on small sample datasets. Although Transformer-based architectures have recently demonstrated strong capabilities in capturing long-range dependencies and modeling global context, they are often associated with higher model complexity and demand more data and computational resources. In contrast, U-Net and its improved variants maintain a relatively low number of parameters and efficient inference speed, while still achieving sufficient segmentation accuracy for the eyelid and corneal regions in this study. Therefore, we adopt the U-Net architecture as the core network structure to ensure both segmentation performance and practical training efficiency for our system. This study proposes an Attention Double U-Net (AtDU-Net) segmentation method to achieve precise segmentation of both the corneal and palpebral fissure regions simultaneously.

**Figure 2:**
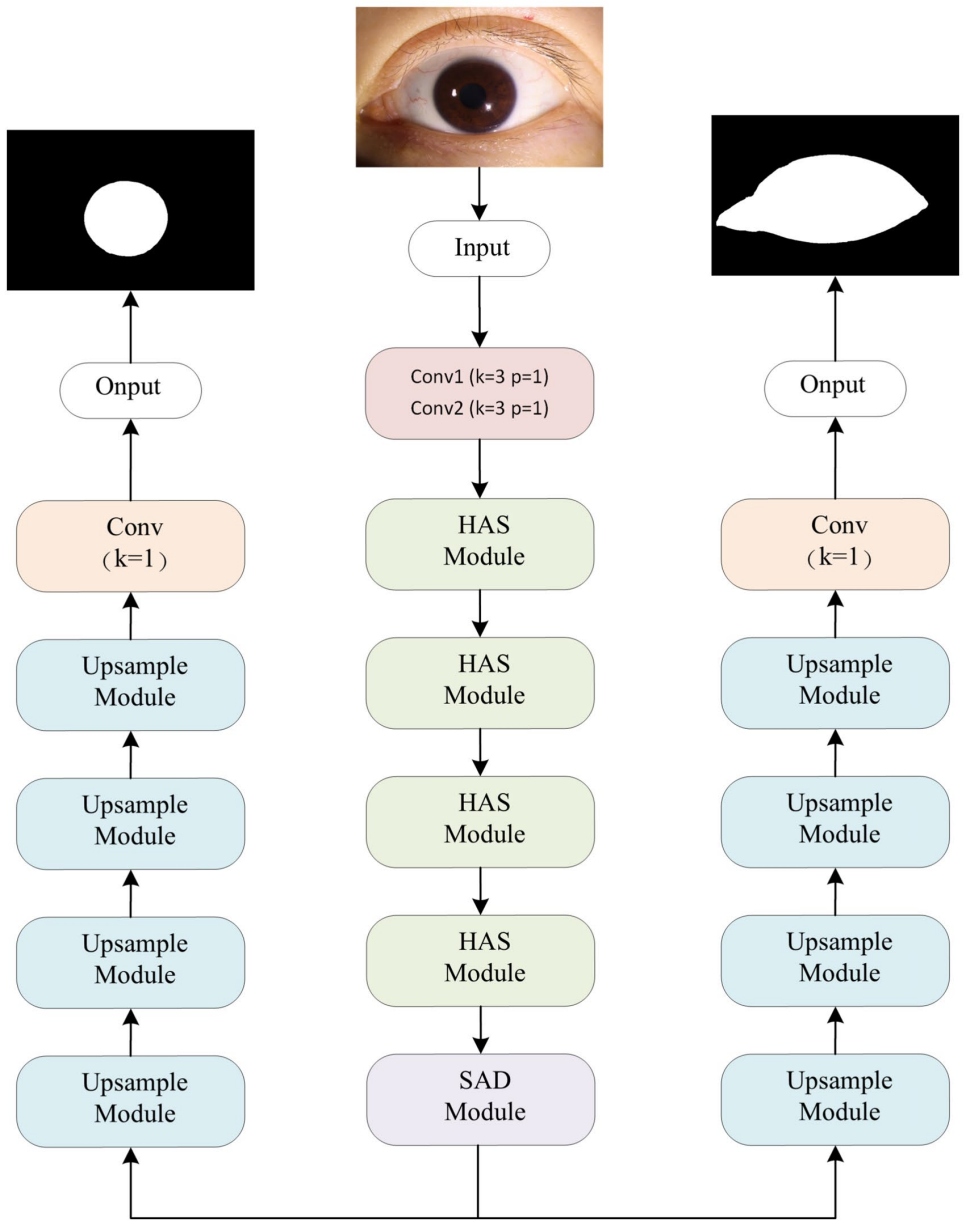
Attention double U-Net segmentation network architecture.

The overall structure of the segmentation network is shown in Figure 2, consisting of a shared feature extraction backbone network and dual decoders. The shared feature extraction backbone network is composed of two convolutional layers, three Hierarchical Attention Sampling Modules (HASM), and Split Axial Detail Modules (SADM). The feature extraction backbone network adopts a hierarchical shared design, which extracts multi-level feature information at different scales through cascaded convolution operations, enabling the capture of complex structural features in both the palpebral fissure and corneal regions. This design not only enhances the model’s ability to capture the complexity of the input images and subtle regional differences, but also significantly improves the efficiency of feature extraction for both regions. The hierarchical shared feature extraction mechanism thus lays a solid foundation for both the efficiency and accuracy of the segmentation task.

To further meet the specific requirements of corneal and palpebral fissure regions in the segmentation task, the network introduces a symmetric dual-decoder structure, which independently decodes the corneal and palpebral fissure regions. In traditional single-decoder architectures, the inability to fully capture the characteristics of different regions often leads to confusion of feature information, thereby affecting segmentation accuracy. The dual-decoder design, through the independence of the decoding paths, ensures that the corneal and eyelid regions do not interfere with each other during the segmentation process. Specifically, each decoder can adopt a more refined decoding strategy based on the unique characteristics of its target region, addressing differences in shape, boundaries, and feature distribution. This independent decoding approach significantly enhances the adaptability of the segmentation model, enabling it to optimize segmentation for the distinct characteristics of each region. This not only improves segmentation accuracy but also significantly enhances the model’s generalization ability for multi-region segmentation tasks.

Therefore, the dual U-Net architecture, by incorporating a shared feature extraction backbone and dual-decoder structure, ensures the relative independence of region-specific segmentation tasks while simultaneously enhancing the overall robustness and operational efficiency of the segmentation system.

#### 2.2.2 Hierarchical attention-based adaptive sampling

In deep learning tasks for image segmentation, feature extraction is one of the key factors determining model performance. However, traditional convolution operations have certain limitations in capturing global contextual information and modeling multi-scale features, often failing to meet the high precision requirements for detailed features in complex scenarios. Specifically, the local receptive field of convolution operations makes it difficult for the model to comprehensively capture global information when handling cross-region dependencies, which is particularly disadvantageous for image features with blurry boundaries or significant scale differences. Additionally, the importance of feature channels often varies significantly in practical applications, and a single, fixed sampling strategy cannot flexibly highlight the feature expression of key regions, leading to limited performance in parsing complex details and ultimately affecting the accuracy of segmentation or detection.

**Figure 3:**
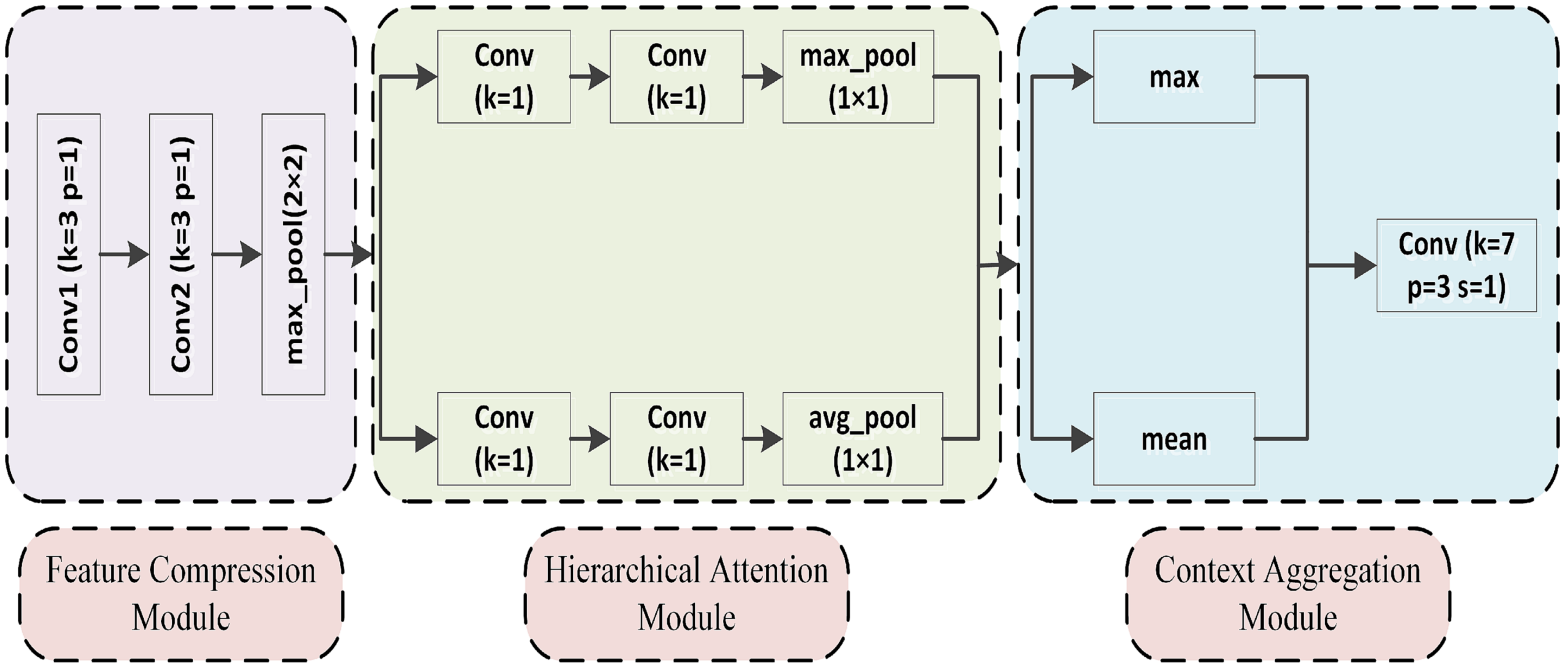
Structure of hierarchical attention sampling module.

To address the issues mentioned above, this study proposes the Hierarchical Attention Sampling Module (HASM), aimed at comprehensively improving the model’s feature extraction ability in key regions. The module consists of three main components: the Feature Compression Module, the Hierarchical Attention Module, and the Context Aggregation Module, as shown in Figure 3. First, the Feature Compression Module extracts key information from the input features through downsampling and local convolution operations, which reduces the computational load while retaining significant local features. Then, the Hierarchical Attention Module adapts the importance of each feature channel through convolution and pooling operations, effectively highlighting the feature expression of key regions while diminishing the influence of redundant or irrelevant features. Finally, the Context Aggregation Module integrates global contextual information into the feature space using a fusion strategy of global max and average pooling, combined with a large kernel convolution operation, which further enhances the model’s ability to perceive interactions between distant regions. By leveraging its advantages in feature selection and enhancement, HASM significantly improves the model’s ability to recognize detailed boundaries, especially when dealing with complex scenes and blurry boundaries. Its hierarchical attention mechanism and multi-scale feature integration design ensure high-quality segmentation results. Additionally, in terms of computational efficiency, the effective combination of downsampling and attention mechanisms significantly reduces computational complexity while maintaining high-resolution features, thereby enabling efficient feature extraction and processing and improving computational resource utilization. These improvements collectively enhance the model’s overall performance in segmentation tasks, providing strong support for feature extraction in the eyelid and corneal segmentation tasks.

#### 2.2.3 Attention mechanism

**Figure 4:**
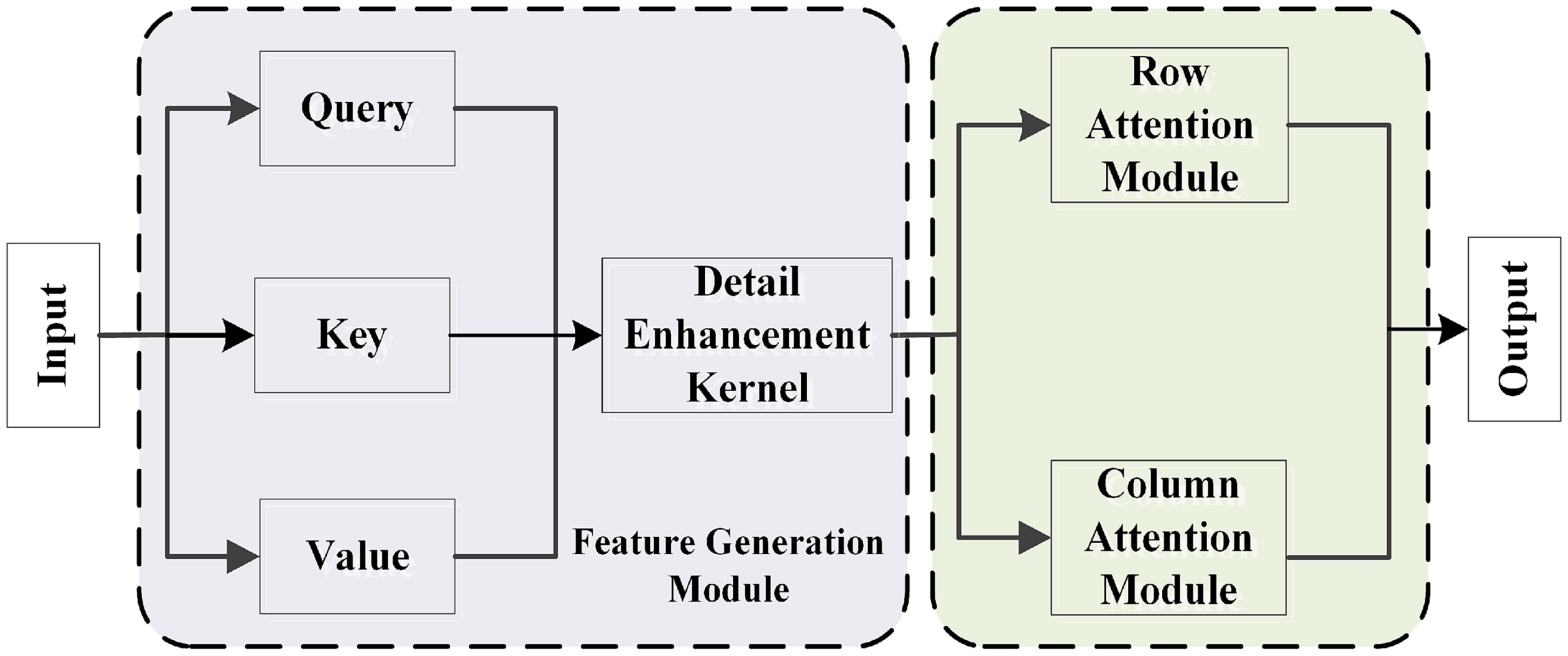
Structure of split axial detail module.

In deep learning tasks for image segmentation, efficiently capturing key features and modeling both global and local dependencies is one of the core challenges to improving model performance. To address this challenge, this study proposes the Split Axial Detail Module (SADM), which aims to significantly enhance the model’s ability to model multi-scale features and capture boundary details through innovative feature compression and axial attention mechanisms. The SADM module consists of three key components: the Feature Generation Module, the Row Attention Module, and the Column Attention Module, as shown in Figure 4. First, the Feature Generation Module refines the input features through local convolution operations, strengthening feature detail expression and local dependency modeling. Then, the features undergo compression and modeling through the Row Attention Module and Column Attention Module, respectively. The Row Attention Module calculates global dependencies in the row direction, enhancing feature coherence in the vertical direction, while the Column Attention Module focuses on dependency modeling in the column direction, thereby improving feature representation in the horizontal direction. The synergistic effect of row and column attention allows the module to fully capture global feature information from different directions while maintaining the diversity and integrity of feature expressions.

The SADM module, by splitting feature modeling into row and column directions, significantly enhances the feature expression capability while effectively reducing computational complexity. Additionally, by combining the Feature Generation Module with the Attention Modules, the model is able to retain key detailed features while enhancing its ability to perceive long-range dependencies, thereby enabling precise segmentation of target regions even in complex scenarios. Through the use of SADM, this study has significantly strengthened the model’s global modeling capability and ability to capture detailed features, providing crucial technical support for multi-scale feature integration and region dependency modeling.

#### 2.2.4 Measurement module

**Figure 5:**
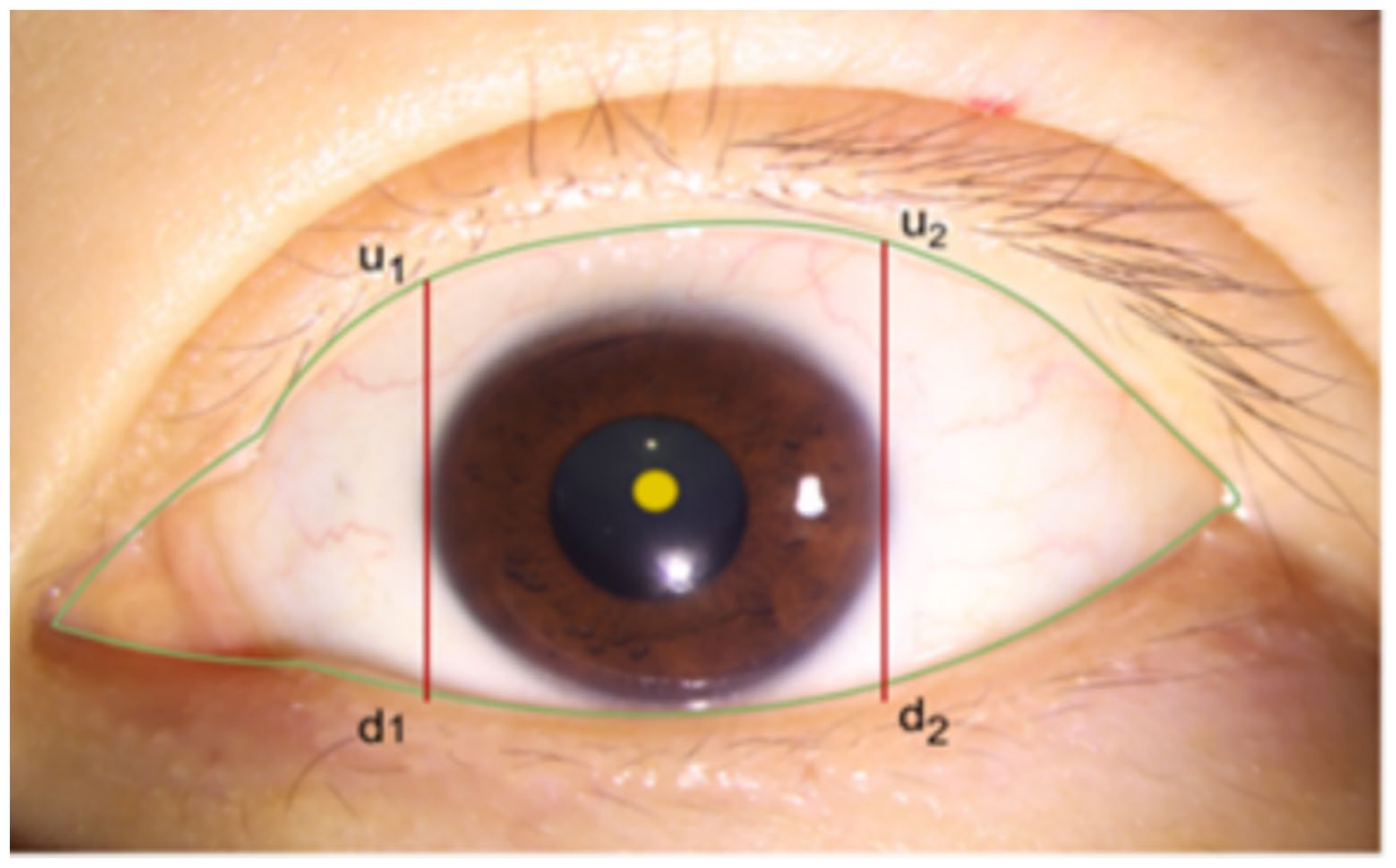
Schematic diagram of eyelid line curvature measurement range.

After segmenting the palpebral fissure and corneal regions, we further implement the measurement of the curvature values for the upper and lower eyelid lines. Upon closely observing a large number of eyelid contour images, we found that the shape changes at both ends of the eyelid are more pronounced and prone to abrupt variations, while the curvature in the middle part is relatively stable and more accurately reflects the state of the eye. Based on professional advice from ophthalmologists, we decided to focus only on the middle portion of the upper and lower eyelid lines for curvature calculation. Specifically, as shown in Figure 5, using the cornea as a reference, we define the intersection points of the tangents at the far-left and far-right edges of the cornea with the upper and lower eyelid lines as $u_{1}$ and $d_{1}$ , and $u_{2}$ and $d_{2}$ , respectively. Then, by calculating the average curvature of all points along the upper eyelid line within the segment from $u_{1}$ to $u_{2}$ , we represent the overall curvature of the upper eyelid line; similarly, by calculating the average curvature of all points along the lower eyelid line within the segment from $d_{1}$ to $d_{2}$ , we represent the overall curvature of the lower eyelid line.

The specific operation process for the measurement is as follows:

- Obtain the Palpebral Fissure Edge Line: Perform edge detection on the segmented palpebral fissure region to identify and extract its edge line. The pixel values along the edge line are set to 1, while the background pixels are set to 0. Starting from the bottom left corner of the image as the coordinate origin, traverse the edge detection results and record the coordinates of all pixels along the palpebral fissure edge line.
- Circle Fitting for the Corneal Region: For the segmented corneal region, fit the smallest enclosing circle to calculate the center coordinates c(m,n) and the radius r of the cornea.
- Obtain the Intersection Points of the Upper and Lower Eyelid Lines: Using the coordinate data of the palpebral fissure edge line and the center and radius of the cornea, determine the corneal region’s horizontal coordinate range as [m−r,m+r] . By detecting the edge pixels at the range endpoints m−r and m+r , find the intersection points of the tangents on both sides of the cornea with the upper and lower eyelid lines. This step gives the upper and lower eyelid line portions within the horizontal coordinate interval [m−r,m+r] . The coordinates of these points are then fitted to a quadratic curve $y=ax^{2}+\mathrm{bx}+c$ .
- Curvature Calculation: Curvature Calculation: For a smooth planar curve defined as $y=ax^{2}+\mathrm{bx}+c$ , the curvature K at a given point is mathematically expressed as:
$$K=\frac{1}{r}=\frac{|y\prime \prime |}{{\left(1+{\left(y\prime \right)}^{2}\right)}^{\frac{3}{2}}}$$
  Where r denotes the radius of curvature, y′ is the slope of the tangent line at the point, and y″ is the second derivative representing the rate of change of the slope (i.e., the local bending). The denominator ${\left(1+{\left(y\prime \right)}^{2}\right)}^{\frac{3}{2}}$ serves as a normalization factor under unit-speed arc length parameterization, ensuring that the curvature reflects intrinsic geometric properties of the curve. A larger value of K indicates a sharper turn or higher bending intensity at that point on the curve.
- Calculate Eyelid Curvature: Based on the center coordinates c(m,n) and radius r of the cornea, the curvature values of the upper and lower eyelid lines can be calculated using the fitting functions of the eyelid lines within the horizontal coordinate range [m−r,m+r] .

**Figure 6:**
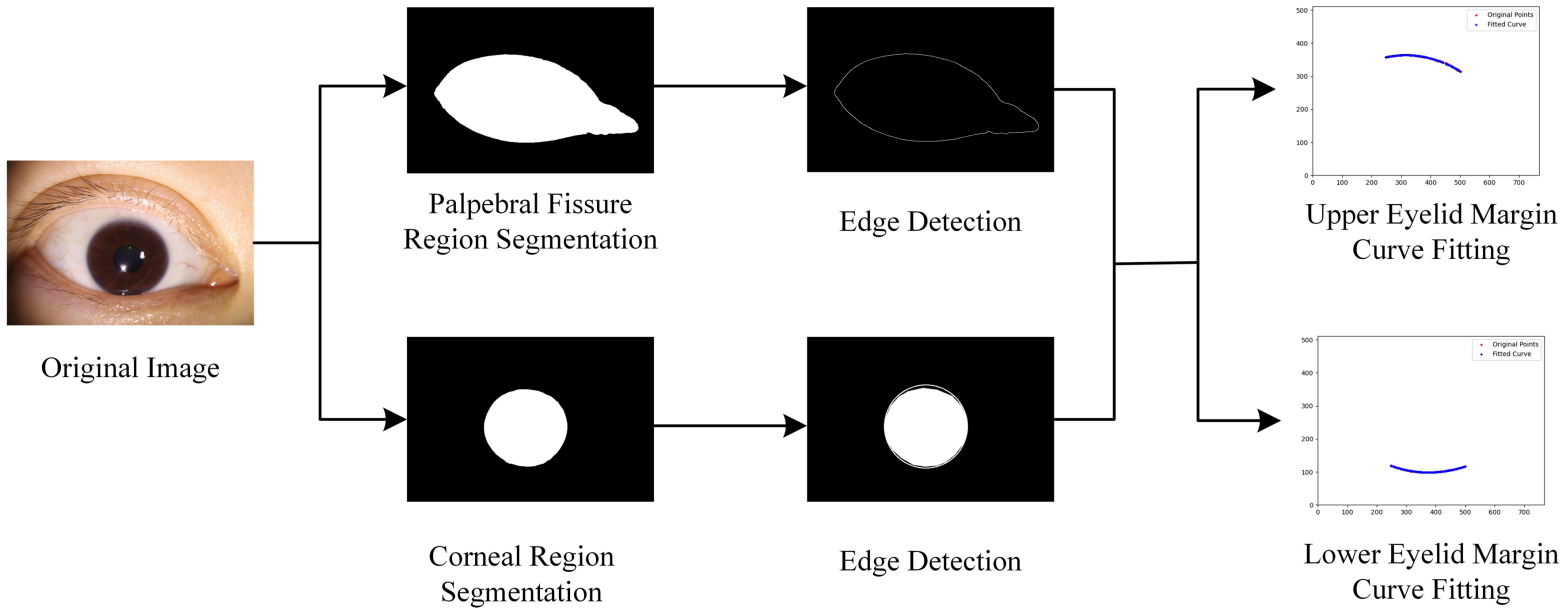
Overall process diagram of eyelid line curvature measurement.

The overall process and details of the eyelid line curvature measurement are shown in Figure 6.

## 3 Experiments and results

### 3.1 Segmentation results

This study used the AtDU-Net network based on U-Net for segmenting the palpebral fissure and corneal regions. The detailed segmentation evaluation results are shown in Table 1. To assess the performance of the segmentation network, this study compared multiple classic networks through experimental validation. The comparative results of the segmentation performance of different models are shown in Table 2. From the segmentation results in Table 2, AtDU-Net demonstrated significant advantages in segmentation performance, with stronger performance in boundary consistency and detail preservation. However, the IoU values of AtDU-Net and Unet++ were the same, which may be because when the model’s segmentation capability has already reached a high level in capturing the overall region, further improvement in IoU values could be constrained by data characteristics and evaluation standards. Moreover, the scale of the dataset and the precision of annotations could affect the differences in IoU. In this context, although there was no significant improvement in IoU, AtDU-Net exhibited higher consistency and boundary recognition capabilities in the Dice coefficient through superior edge feature processing. Table 3 presents the segmentation results for the palpebral fissure and corneal regions.

**Table 1:** Segmentation evaluation metrics for palpebral fissure and corneal regions.

Region	IoU	Dice coefficient	HD95	ASSD
Palpebral fissure region	0.980	0.990	9.120	2.309
Corneal region	0.974	0.987	7.975	2.695

**Table 2:** Quantitative performance comparison of different models.

Models	IoU	Dice coefficient
U-Net	0.972	0.985
Attention U-Net	0.975	0.987
Unet++	0.979	0.988
Swin-UNet	0.973	0.983
TransUNet	0.978	0.986
AtDU-Net	**0.979**	**0.989**

Bold values indicate the segmentation results of the proposed method.

**Table 3:** Examples of segmentation results for palpebral fissure and corneal region.

Original image	Palpebral fissure mask	Palpebral fissure prediction	Corneal mask	Corneal prediction
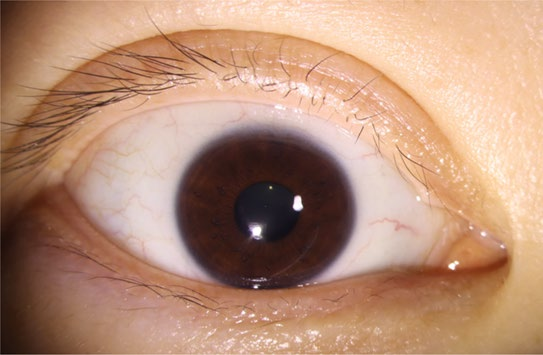	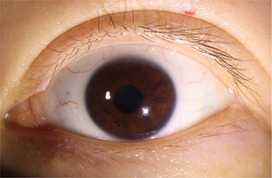	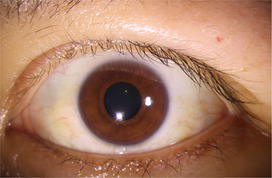	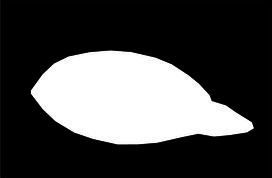	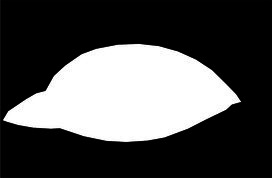
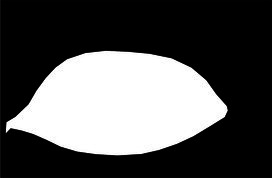	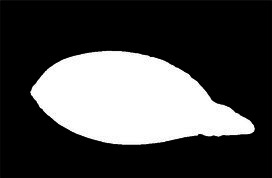	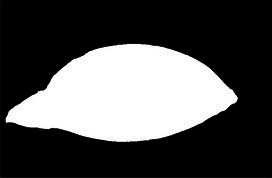	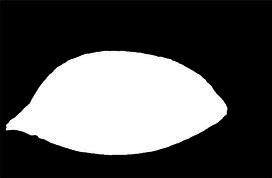	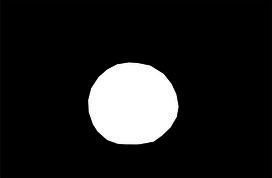
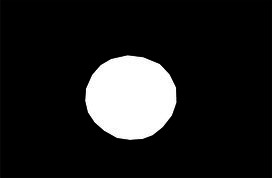	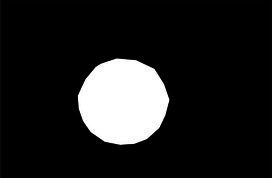	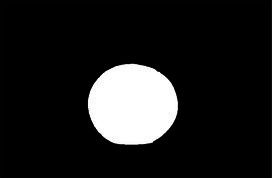	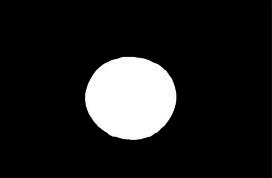	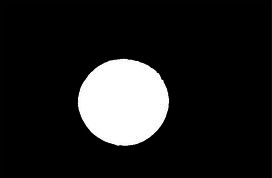

To further elucidate the structural advantages of AtDU-Net, a series of ablation experiments were systematically designed and conducted by selectively removing or combining key modules. The experimental results are presented in Table 4. It can be observed that introducing the HAS module into the baseline network (Basenet) significantly improves both the Dice coefficient and IoU metrics, primarily due to the module’s enhanced capability in capturing fine-grained local details. Similarly, incorporating the SAD module effectively boosts segmentation performance by strengthening global feature modeling. When the HAS and SAD modules are integrated simultaneously, a complementary relationship between local detail extraction and global contextual modeling is established, leading to further performance gains. Moreover, when segmentation is performed separately on either the palpebral fissure region or the corneal region, the overall network performance declines. In contrast, jointly segmenting both regions enhances the complementarity within the feature space and improves context awareness, thereby achieving more accurate boundary localization and region separation.

**Table 4:** Ablation study results.

BaseNet	HAS module	SAD module	Palpebral fissure region	Corneal region	IoU	Dice coefficient
√					0.963	0.977
√	√				0.979	0.981
√		√			0.973	0.978
√	√	√	√		0.971	0.984
√	√	√		√	0.970	0.983
√	√	√	√	√	**0.979**	**0.989**

Bold values indicate the segmentation results of the proposed method.

### 3.2 Curvature measurement results

**Figure 7:**
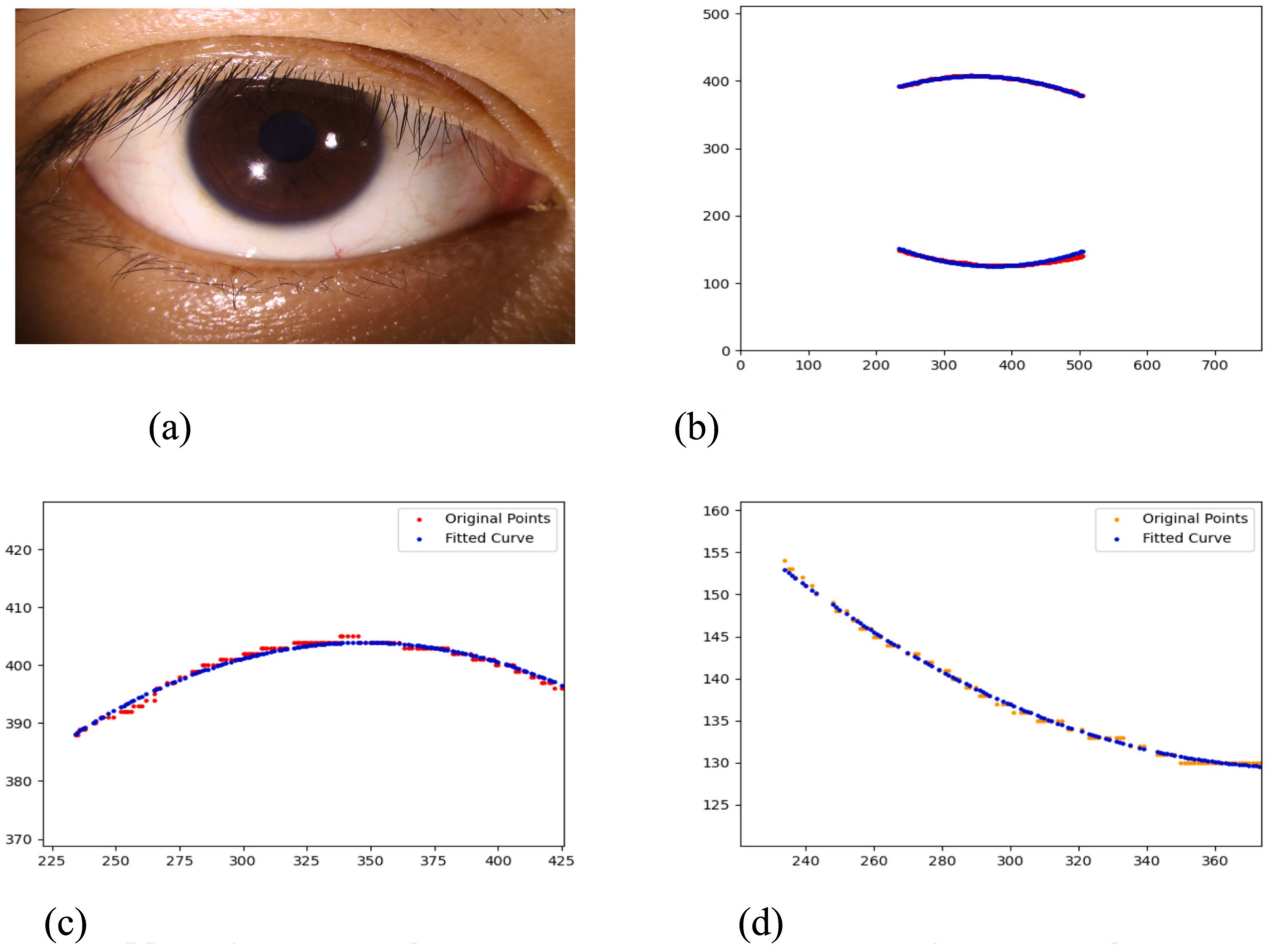
Detailed fitting of upper and lower eyelid lines. **(a)** Original image **(b)** Fitted upper and lower eyelid margin curves **(c)** Detailed fitting of the upper eyelid margin curve **(d)** Detailed fitting of the lower eyelid margin curve in addition, the titles of the figures have been revised to detailed eyelid margin curve fitting for upper and lower eyelids.

After segmenting the palpebral fissure and corneal regions, the next step is to fit the upper and lower eyelid lines, as shown in Figure 7, which includes four images that demonstrate the complete process of fitting the target upper and lower eyelid lines. In these images, red dots represent the original coordinates of the upper eyelid line, orange dots represent the original coordinates of the lower eyelid line, and blue dots indicate the coordinates of the fitted upper and lower eyelid lines. Specifically, after performing Canny edge detection on the palpebral fissure region, a coordinate system is established with the bottom-left corner of the image as the origin. This coordinate system is then used to provide the reference for fitting the upper and lower eyelid lines. Using this coordinate system, the list of coordinates for all points on the upper and lower eyelid lines is accurately extracted. To avoid cases where multiple vertical coordinates correspond to the same horizontal coordinate, the coordinate list is converted into a set to ensure that each horizontal coordinate corresponds to a unique vertical coordinate. This study uses the coordinates of the pixel points to fit the upper and lower eyelid lines. Unlike traditional methods, the fitting approach proposed here does not rely on manually labeled points on the eyelid contour, thus significantly reducing the influence of subjectivity. This method not only improves the accuracy of the fitting results but also greatly enhances the reproducibility of the measurement outcomes.

Table 4 presents the curvature values of the target upper and lower eyelid lines measured manually and automatically. Table 5 shows the $r^{2}$ for the curvature measurements of the target upper and lower eyelid lines using both methods, with all $r^{2}$ exceeding 0.9. This indicates a high degree of consistency between the automatic and manual measurements, demonstrating that the automatic measurement method has high reliability for practical applications. Table 6 presents the mean absolute error (MAE) and root mean square error (RMSE) between the automatic and manual measurements. The results indicate that the automatic measurement method performs excellently in terms of accuracy, with small errors, demonstrating high practical value and reliability.

**Table 5:** Curvature measurement results for target upper and lower eyelid margin curves using manual and automatic methods.

Measurement method	Upper eyelid line curvature (*mm^-1^*)	Lower eyelid line curvature (*mm^-1^*)
Manual measurement	0.247 ± 0.005	0.203 ± 0.007
Automatic measurement	0.242 ± 0.005	0.197 ± 0.006

**Table 6:** Error comparison between manual and automatic curvature measurements of upper and lower eyelid margin curves.

Accuracy indicators	Upper eyelid	Lower eyelid
MAE	0.0075	0.0068
RMSE	0.0122	0.0090

Bold values indicate the segmentation results of the proposed method.

**Figure 8:**
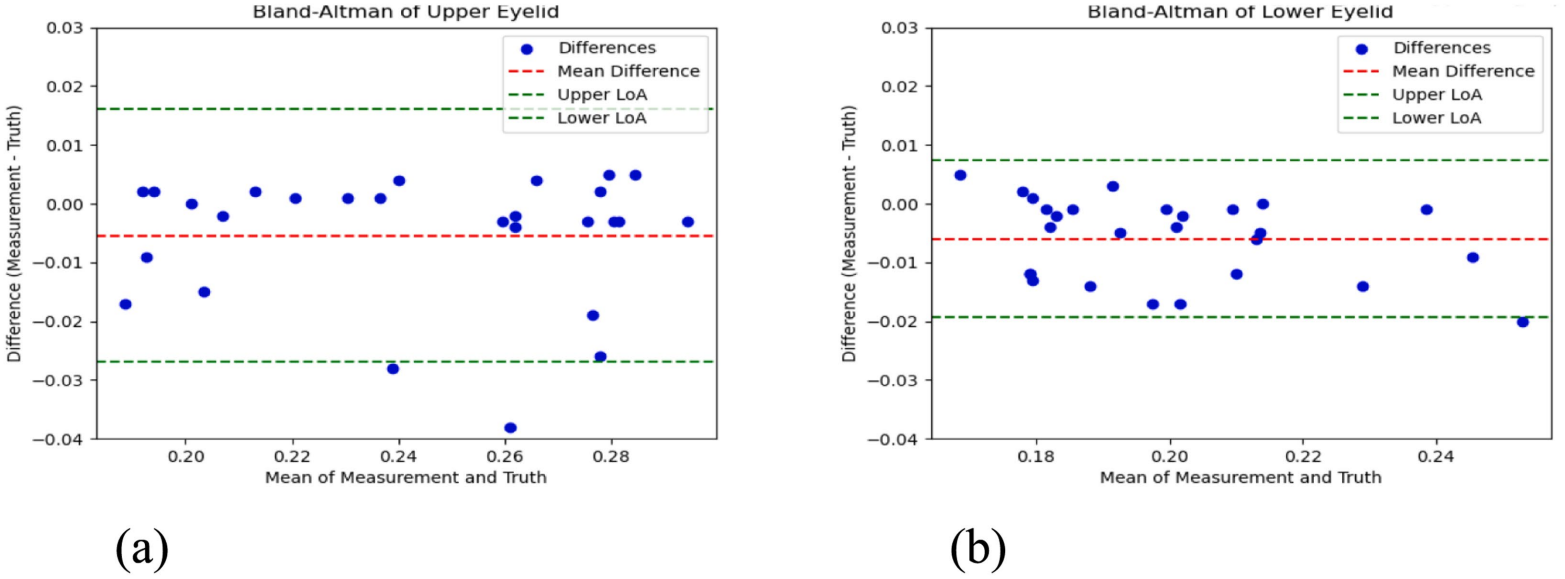
Bland–Altman plot of upper and lower eyelid curvature in the test set. **(a)** Bland-Altman plot for upper eyelid curvature **(b)** Bland-Altman plot for lower eyelid curvature.

The Bland–Altman plots are shown in Figure 8. The x-axis of the Bland–Altman plot represents the mean of automatic and manual measurements, while the y-axis represents the difference between the automatic and manual measurements. Specifically, (a) is the Bland–Altman plot for the upper eyelid curvature, and (b) is the Bland–Altman plot for the lower eyelid curvature. From the Bland–Altman plots, it can be observed that the upper eyelid curvature plot contains 27 data points, with 25 points falling within the limits of agreement, accounting for 92.6%. Similarly, the lower eyelid curvature plot contains 27 data points, with 26 points falling within the limits of agreement, accounting for 96.3%. This confirms that the automatic and manual measurements show good consistency in the analysis of both upper and lower eyelid curvatures. Moreover, the narrow limits of agreement reflect small measurement errors, indicating that the measurement system has high accuracy and robustness.

**Figure 9:**
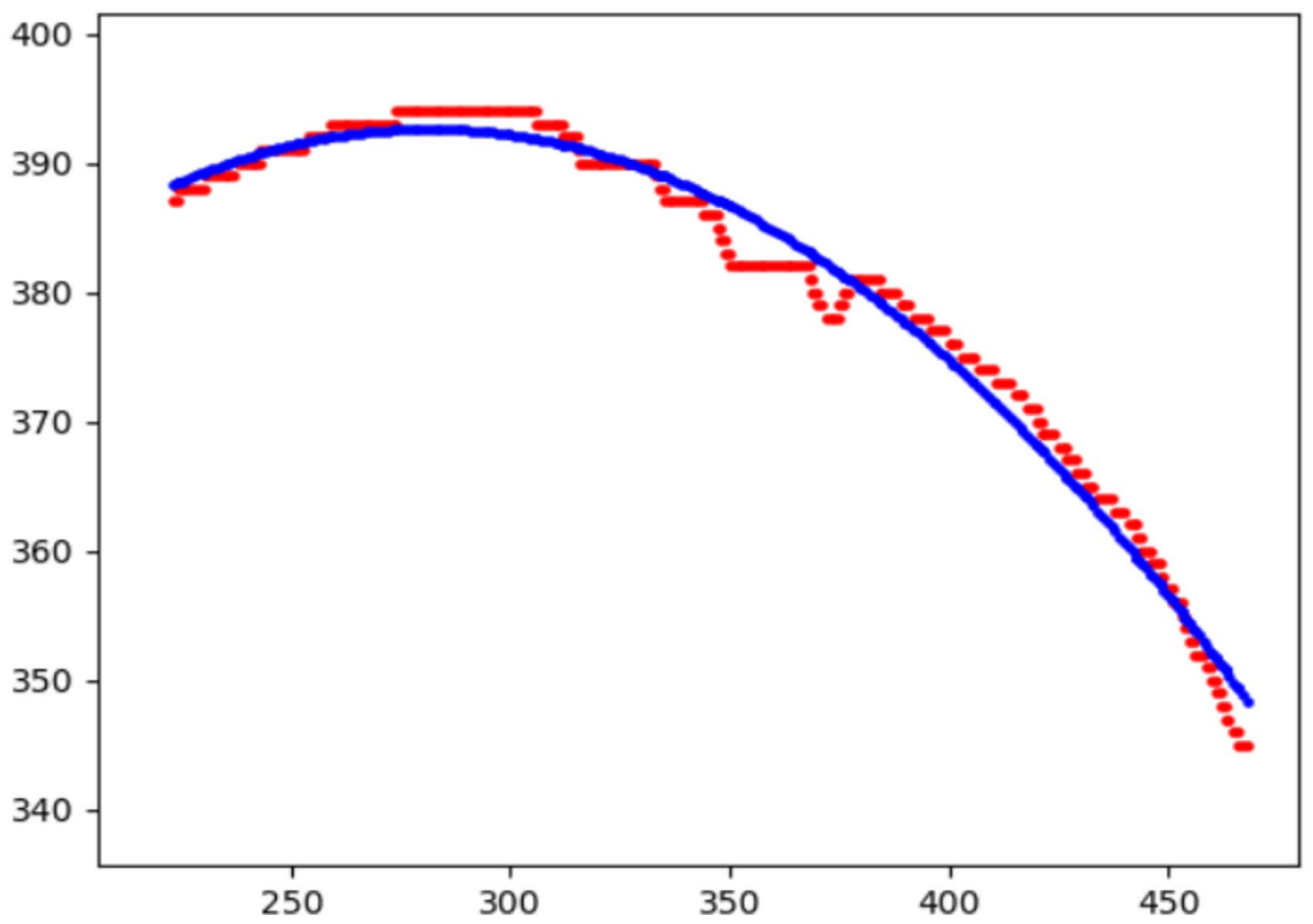
Fitted curvature curves at key positions.

To further validate the robustness of our method under exceptional conditions, this study takes an upper eyelid test sample as an example. We calculated the curvature at three key positions ( m−r , m , m+r ,) based on both automatic measurements and manual annotations, with the fitting results shown in the Table 7 and the fitted images shown in Figure 9. We found that the main source of error arises from slight pixel-level deviations along the predicted mask boundary, which affect the extraction of edge points. Since curvature calculation is highly sensitive to local variations of the fitted boundary, such deviations may lead to amplified curvature differences. In future work, we plan to incorporate mechanisms for outlier detection and analysis to further enhance the model’s robustness and practical applicability.

**Table 7:** Comparison of curvature values at key positions between automatic measurements and manual annotations.

Measurement method	*m-r*	*m*	*m+r*
Manual measurement	0.286	0.291	0.197
Automatic measurement	0.267	0.265	0.188

To comprehensively display the morphological characteristics of the eyelids, this study selects six representative feature points on the upper and lower eyelid lines for curvature measurement. These points are located at the intersections of the upper and lower eyelid lines with three vertical lines: one at the tangent points on both sides of the cornea and two passing through the center of the cornea. The intersection closer to the outer canthus is referred to as the left point, denoted as $U_{l}$ ; the intersection passing through the center of the cornea is referred to as the center point, denoted as $U_{m}$ ; and the intersection closer to the inner canthus is referred to as the right point, denoted as $U_{r}$ . In the curvature analysis of these feature points, Tables 6, 8 present the fitting results of the upper and lower eyelid lines, along with their corresponding feature points’ manually and automatically measured curvature values. In these tables, the first value in each row of the last three columns represents the manually measured result, while the second value represents the automatically measured result. This method allows for a direct comparison between manual and automatic measurements of curvature at each feature point, thus verifying the accuracy and robustness of the automatic measurement system (Table 9).

**Table 8:** Consistency between manual and automatic curvature measurements of upper and lower eyelid margin curves.

Fit consistency	Upper eyelid line	Lower eyelid line
$r^{2}$	0.9032	0.9154

**Table 9:** Curvature test results of upper eyelid feature points.

Original image	Upper eyelid margin curve fitting	*U_l_*	*U_m_*	*U_r_*
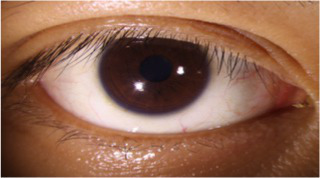	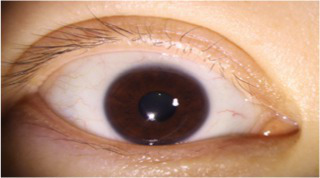	0.212 0.214	0.236 0.237	0.193 0.195
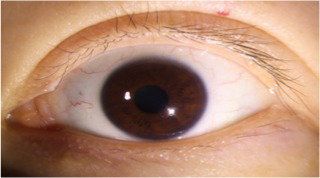	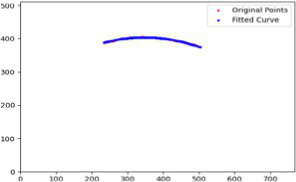	0.261 0.258	0.263 0.261	0.191 0.193
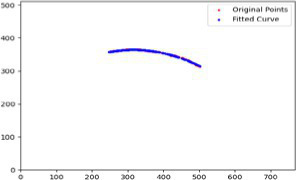	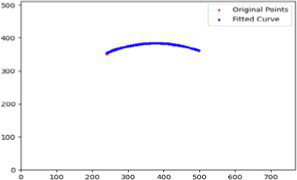	0.230 0.231	0.282 0.287	0.238 0.242

## 4 Discussion

This study proposes a deep learning-based automated eyelid line curvature measurement method, which achieves efficient measurement of the upper and lower eyelid line curvatures through precise segmentation of the palpebral fissure and corneal areas, as well as eyelid margin curve fitting of the target eyelid lines (Table 10). This method not only significantly reduces the workload of manual intervention but also overcomes the limitations of traditional methods, providing a standardized and repeatable solution for precise ocular surface morphology analysis. It also offers reliable data support for ophthalmic clinical diagnosis and research. From the segmentation results, the designed AtDU-Net model achieved an IoU of 0.979 and a Dice coefficient of 0.989, significantly outperforming the traditional U-Net and its variants, fully validating the proposed model’s efficiency and accuracy in segmentation tasks. As for curvature measurement, the correlation between automatic and manual measurement results reached 0.9032 for the upper eyelid line and 0.9154 for the lower eyelid line. Combined with the Bland–Altman analysis, the measurement errors for most points fall within the limits of agreement, further demonstrating the advantages of the proposed automatic measurement method in terms of accuracy and robustness.

**Table 10:** Curvature test results of lower eyelid feature points.

Original image	Lower eyelid margin curve fitting	*U_l_*	*U_m_*	*U_r_*
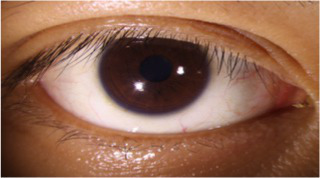	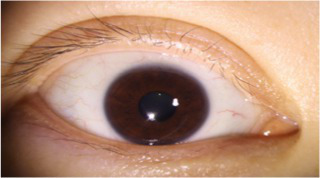	0.166 0.171	0.190 0.193	0.177 0.179
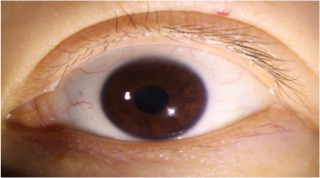	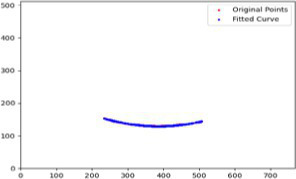	0.216 0.210	0.250 0.241	0.216 0.211
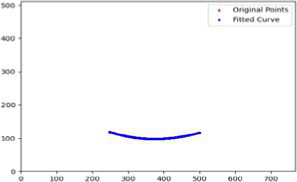	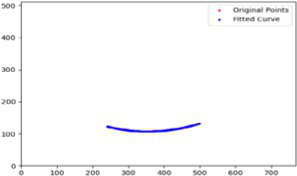	0.214 0.214	0.239 0.238	0.203 0.201

Compared to traditional curvature measurement methods that rely on manual annotation, this study significantly improves measurement efficiency and achieves breakthroughs in accuracy and consistency through the organic combination of automatic segmentation and eyelid margin curve fitting. In terms of segmentation, the proposed AtDU-Net network, with its dual U-shaped decoder structure and hierarchical feature-sharing mechanism, effectively meets the specific demands of corneal and palpebral fissure regions in segmentation tasks. Traditional segmentation models often struggle to handle the unique features of multiple anatomical regions simultaneously. However, the decoder design of AtDU-Net achieves precise capture of multi-region features in complex scenarios by creating separate decoding paths for the corneal and palpebral fissure regions. Additionally, the HASM and SADM modules further enhance the network’s performance. The HASM module excels in multi-scale feature extraction and focusing on key regions, effectively strengthening attention on detailed areas, while the SADM module plays a critical role in global context modeling, allowing the network to balance global information with local details. This ensures that the network maintains high-precision segmentation even in complex scenarios. These improvements not only provide more reliable input for subsequent eyelid margin curve fitting but also lay the foundation for the robustness of the overall segmentation process.

In terms of eyelid margin curve fitting, this study overcomes the shortcomings of traditional manual annotation. The automated eyelid margin curve fitting process, combined with segmentation results and pixel point coordinate extraction, allows for precise capture of the geometric features of the upper and lower eyelid lines. This effectively eliminates the inaccuracies in curvature calculation caused by errors in manual operation details. Moreover, compared to traditional methods that rely on manually annotated points, this study employs a fully automated eyelid margin curve fitting strategy, enhancing the accuracy and repeatability of the measurements.

However, this study still has certain limitations. First, the dataset used is relatively small. Although the model shows strong generalization ability, its performance still needs further validation on larger and more diverse datasets. Second, for curvature measurement, only the feature points in the middle section of the upper and lower eyelid lines were selected for calculation, and a fine-grained analysis of the entire eyelid curve was not performed. Future research could focus on the following directions: On one hand, introducing larger-scale and more diverse annotated datasets along with transfer learning techniques can further improve the model’s robustness and universality. On the other hand, combining other fitting algorithms to conduct a more in-depth study of the overall shape of the eyelid curve could expand the model’s application scenarios in ophthalmic clinical practice.

## 5 Conclusion

This study proposes an automated eyelid line curvature measurement method based on deep learning, which integrates a precise segmentation model with an efficient curvature measurement technique. In the segmentation task, the proposed AtDU-Net model combines a shared feature extraction backbone network with a dual-decoder structure, significantly improving segmentation efficiency while maintaining high accuracy. Based on the segmentation results, the measurement module employs a pixel-point eyelid margin curve fitting method, effectively enhancing the accuracy of the automatically measured curvature values. Compared with traditional curvature measurement methods that rely on manual annotations, this non-invasive system not only improves measurement accuracy but also reduces manual intervention and the influence of subjective factors, greatly enhancing efficiency and repeatability. It holds significant value for the diagnosis, treatment, and postoperative evaluation of eyelid-related diseases.

## Data Availability

The raw data supporting the conclusions of this article will be made available by the authors, without undue reservation.
